# A description of the use of zuclopenthixol decanoate long-acting injection in a large mental health trust

**DOI:** 10.1192/bjo.2021.891

**Published:** 2021-06-18

**Authors:** Shay-Anne Pantall, Emily Whitehouse, Lisa Brownell

**Affiliations:** 1 Birmingham and Solihull Mental Health NHS Foundation Trust; 2 University of Birmingham, Medical School

## Abstract

**Aims:**

Adherence with antipsychotic medication is an important factor in the prevention of relapse in psychotic disorders such as schizophrenia. Long acting antipsychotic injections promote improved adherence. In recent years, second generation antipsychotic long-acting injections have become increasingly popular, and little has been written about the use of the older depot medications. Here, we explore the current use of one of the first-generation antipsychotic long acting injections in Birmingham and Solihull Mental Health NHS Foundation Trust.

**Method:**

An 18-month retrospective case-note review of all patients who started zuclopenthixol decanoate during the first 6 months of 2018 (n = 45)

**Result:**

Key findings included: -

- 71% were male
- The mean age was 37 (range 19-65)
- The most common diagnoses were: schizophrenia (51%), bipolar affective disorder (18%) and schizoaffective disorder (13%). We noted that 2 individuals (4%) had a primary diagnosis of recurrent depressive disorder, 2 (4%) had a primary diagnosis of emotionally unstable personality disorder.
- 60% of those who were prescribed zuclopenthixol decanoate discontinued it within the 18-month follow-up period.
- The vast majority of discontinuation occurred within the first 6 months, and after this, few individuals stopped treatment.
- The most common reason for discontinuation was side effects (57%), with other reasons including patient choice (7%), non-compliance (7%), pregnancy (4%), or needle phobia (4%).

**Conclusion:**

Zuclopenthixol decanoate has been used for individuals with both schizophrenia and paranoid psychosis (where it is licenced) and also occasionally for other indications. A high proportion discontinued the zuclopenthixol within 6 months, this generally being attributed to adverse effects. Those who were still receiving this medication at 6 months were very likely to continue to take it throughout the 18 months. We would therefore recommend robust monitoring for and management of adverse effects in the early phases of treatment.

